# Knowledge and Acceptance of COVID-19 Vaccine: A Community-Based Cross-Sectional Study Among Residents of Jinja District Eastern Uganda

**DOI:** 10.24248/eahrj.v8i2.786

**Published:** 2024-06-26

**Authors:** Hawa Mutesi, Nurhan Meydan Acimis

**Affiliations:** a Pamukkale University-Denizli Turkey.

## Abstract

**Background:**

Despite the existence of other public health interventions, vaccination remains a cornerstone in the fight against pandemics. COVID-19 has led to loss of many lives, global economic deteriorations, and unemployment, hence the call for urgent interventions which includes introduction of COVID-19 vaccines.

**Aim:**

The study aimed to assess the level of knowledge and acceptance of COVID-19 vaccine among residents of Jinja District, Eastern Uganda.

**Method:**

A population-based descriptive cross-sectional study was conducted among 210 participants from Jinja District between 8^th^ and 21^st^ October 2021. Data was collected using a structured questionnaire and analysed using IBM SPSS version 26.

**Results:**

45.2% of the participants had adequate level of knowledge with a 56.2% COVID-19 vaccine acceptability rate. Highly educated participants were most likely to have adequate knowledge of COVID-19 vaccine than the lowly educated ones (OR= 2.64; 95% CI; 1.32-5.26, P= .006). Having a high level of education (OR=2.7; 95% CI; 1.38-5.10, P= .004) was significantly associated with vaccine acceptability. Farmers and students were less likely to accept COVID-19 vaccine.

**Conclusion:**

The general population of Jinja District demonstrated a low level of adequate knowledge and acceptance towards COVID-19 vaccine. There is need for more public awareness campaigns on the topic using radio and television as means of communication.

## BACKGROUND

Corona Virus Disease of 2019 (COVID-19) is a disease caused by a strain of coronavirus known as severe respiratory syndrome-corona virus -2 (SARSCoV-2), which has become a serious public health concern worldwide. The deadly disease originated from the Chinese city of Wuhan in late 2019.^1^ As of today, the SARS-COV-2 virus has affected almost each and every country worldwide due to its rapid mutating and spreading rate. The pandemic has not only destroyed the global economy, but also affected the health system (including routine childhood immunizations) around the world.^2^ Covid-19 was declared a global pandemic by WHO on 11 March 2020.^1^ As of October 30, 2021, there were 245 million cases and 4.98 million deaths worldwide.^3^ There were 8.6 million cumulative cases and 218,000 deaths in Africa.^4^ At the same time, Uganda had 126,075 confirmed cases and 3,215 deaths.^5^ Although the African continent has recorded relatively lower cases of Covid-19 so far, the African Centre for Disease Control and Prevention (CDC) reported a higher case fatality rate in sub-Saharan Africa at 2.5% compared to 2.2% in the rest of the world.^6^ Non-Pharmaceutical Interventions (NPIs) such as wearing face masks, use of hand sanitisers and social distancing need to be implemented globally to slow down the spread of the pandemic. Vaccination provides the best hope for a permanent solution to the control of the pandemic.^6^ However, the acceptance and subsequent uptake of vaccines is stymied by a number of challenges including; vaccine availability and hesitancy. Vaccine hesitancy can be defined as the delay in accepting or rejecting the vaccine despite its availability. There are 3 main factors that influence vaccine hesitancy namely: confidence, convenience, and complacency.^7^ Vaccine hesitancy has been recognised as a serious public health threat and because of this, extensive research should be conducted to fully understand the causes and prevalence of vaccine hesitancy among different populations. In a situation where there is much doubt about the safety and efficacy of the COVID-19 vaccine around the globe, understanding the barriers to vaccine acceptance is very important. To achieve the targeted level of herd immunity, there is need to know the general public's knowledge of the COVID-19 vaccine, its acceptance rate, and what its determinants are.

### COVID-19 Vaccination in the African Region

Epidemiological data have shown that vaccinating a large a large proportion of the population is necessary to achieve herd immunity.^8^ Given that 65% of Africa's population is under the age of 35, the continent faces a lower COVID-19 risk based on age demographics compared to other regions. Consequently, a smaller proportion of the population requires vaccination as a mandatory measure. However, it's crucial to address global inequities in vaccine distribution to ensure a balanced approach.. By October 30, 2021, at least 51% of the global population had been vaccinated with 1 dose of the covid-19 vaccine and 39% had been fully vaccinated. Of these, 1% had received a booster dose.^9^ Although the African continent accounts for 17% of the global population, only 2.5% of the 6.4 billion vaccine doses administered worldwide were administered in Africa.^10^ By the end of September 2021, only 15 African countries (out of 54) had met WHO's target of vaccinating 10% of the general population. Half of the countries on the African continent had fully vaccinated less than 2% of their general population.^11^ This is believed to have resulted from inequalities in vaccine supply, with most African regions being left at the back of the vaccine delivery queue.^10^ In addition, the African continent is also faced with vaccine hesitancy concerns.^11^

### The Situation in Uganda

Uganda launched its COVID-19 vaccine program with the AstraZeneca vaccine on March 10, 2021. Priority was given to healthcare workers, security personnel, teachers, humanitarian frontline workers, and the elderly with chronic illnesses.^12^ By September 16, 2021, 2,799,920 doses of vaccines from AstraZeneca, Sinovac and Moderna had been received in Uganda. However, some Ugandan citizens were hesitant to get vaccinated. This might be due to the influence of social media, negative campaigns against COVID-19 vaccines, exaggerated concerns about vaccine side effects, and the belief among the public that the disease will be cured by herbal treatments.^13^

So far, there are only 2 population-based studies on COVID-19 vaccine-acceptance in the country. However, none of them intended to know the general population's knowledge towards the COVID-19 vaccine. Both studies were conducted in the western part of the country, and those without internet were excluded since data was collected online. Only one study was conducted after the vaccine was availed in the country. Due to the above-mentioned gaps, as well as the cultural and other differences within the various regions of the country, data obtained from those few studies cannot provide comprehensive information about the knowledge level and acceptance rate of COVID-19 vaccine among the general population of Uganda as a whole and Jinja district in particular. Hence calling for more studies on the subject in other parts of the country. This study aims to assess knowledge and acceptance of COVID-19 vaccine among the general population of Jinja District, Eastern Uganda.

## METHODOLOGY

### Study Design

A population-based descriptive cross-sectional study was conducted among 210 participants between 8^th^ and 21^st^ October 2021. This type of study design was used because it allows large volumes of data to be collected at one point in time, saves time, and is not costly.

### Study Area

The study was conducted in Jinja District. Jinja is located in the eastern part of Uganda, on the northern shores of Lake Victoria, and east of River Nile. It borders Kamuli in the north, Luuka in the east, Buvuma in the south, and Buikwe District in the west. The region is divided into 3 counties, 6 sub-counties, 46 parishes and 381 villages.^14^ According to the 2014 National Census, the estimated population of the district was 471,242 people. The adult literacy rate in Jinja was 80.1% in 2014.^15^ The most important economic activity in the region is agriculture. Cotton, sugarcane and coffee are the main cash crops grown in the region. It is 96 kilometres by road from Kampala, the capital city of Uganda. Zone coordinates: 00 30N, 33 12E. (Latitude: 0.5000; Longitude: 33).^14^

### Study Population

The target group was residents of Jinja District aged 18 years and above. All individuals aged 18 years or older, currently residing in Jinja, and had agreed to participate in the study were included and those under 18 years of age and not residents in the study area were excluded.

### Study Variables

Dependent variables included; knowledge and acceptance of the COVID-19 vaccine. Independent variables included; Demographic characteristics (gender, age, marital status, education level, occupation, and residence), source of information, and reasons for acceptance/hesitancy.

### Sample Size

A sample size of 210 participants was used. It was determined basing on the 30*7 cluster sampling method developed by WHO in 1978 to estimate vaccine coverage in middle- and low-income countries. A 95% Confidence Interval, a 10% percentage estimate of the actual population percentage, and an assumed ratio of 0.5 vaccination coverage were considered.



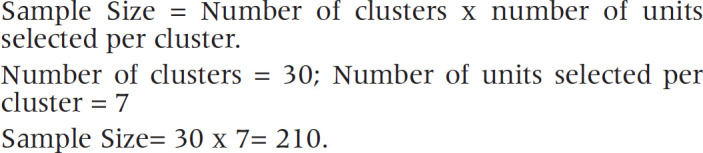



### Sampling Method and Procedure

The 30*7 cluster sampling method was used in the study because it is simple, economical, does not require a list of all population units, and all households in the study area have an equal chance of being included in the sample. The sampling process consisted of two steps: First, 30 clusters (villages) were selected with probability proportional to the most recent census estimate of the village population size by systematic selection from a list of cumulative population sizes; second, 7 households were selected from each of the 30 clusters. In order to select the first household from each village, a random direction was chosen using random numbers generated by the computer from the centre of the village, total number of households from the centre point to the edge of the village in the selected direction were counted and randomly, one of the houses was selected as the starting point. All eligible participants in the household were included in the study after their consent was obtained. From the first household, the next house with the door closest to the first was visited and all eligible participants were included in the study. The process continued until a total of 7 participants were obtained from each village.

### Data Collection Methods and Tools

The study was based on primary data collected directly from the respondents. Data was collected using a structured questionnaire developed based on literature review and discussions within the research team. The questionnaire was developed in English because it is the common language used by the majority in the region. Translation of the questionnaire into local language (lusoga) was done for a few of the participants who did not know English. Socio-demographic, knowledge, and variables related to COVID-19 vaccine acceptance were all included in the questionnaire.

The social demographic characteristics of the participants in the survey were: gender, marital status, education level, occupation and residence.

To estimate the participants' knowledge of COVID-19 vaccine, 10 questions were used with a ‘Yes’ or No answer to each question. A total knowledge score of 0-10 was calculated by giving 1 point to each correct answer and 0 point to each incorrect answer. <5 points score was considered having inadequate knowledge; 5-7, moderate; and 7< was considered having adequate knowledge about COVID-19 vaccination. Participants were also asked how they got information about the COVID-19 vaccine.

Regarding acceptance of the COVID-19 vaccine, participants were asked to answer the following question: Have you been vaccinated/do you want to be vaccinated if the vaccine is available in your community? If the answer is yes/no, another question was asked about why they accepted/rejected the vaccine.

### Data Collection Procedure

Permission to carry out the study was sought from the village heads. Participants' consent was also obtained. Respondents who consented and knew English were immediately issued a questionnaire and requested to give it back to the researcher within 5 to 10 minutes. Verbal translation of the questionnaire from English to lusoga was done for the participants who consented but did not understand English. The researcher helped them by filling in the questionnaire with the responses given.

### Quality Control

The questionnaire underwent preliminary verification by 4 independent reviewers and a pre-test was conducted with 20 individuals to assess its applicability, accuracy and consistency. Responses from the pre-test run were excluded from the final analysis. Concerns about questions in the study questionnaire raised during the pre-test were considered and appropriate changes were made. To streamline the data collection process, the researcher provided training to research assistants on the study's key elements. This approach not only expedited data collection but also minimised potential errors and mitigated any bias that could arise from having a single individual conduct all data collection Collected information was cross checked by the researcher for consistence and completeness.

### Ethical Approval

All participants were informed about the purpose of the study prior to their participation and were assured that their personnel information would not be shared with anyone. The study was approved by Pamukkale University, Non-Interventional Clinical Research Ethics Committee (approval letter No. E-60116787-020-106862; date: 23.09.2021).

### Data Presentation and Analysis

Data was analysed using IBM SPSS software version 26. Sociodemographic information and categorical data on COVID-19 vaccine acceptance are presented as frequency and percentage in text, tables, figures and graphs. The relationship between independent and dependent variables was tested using the Chi-square test. Logistic Regression tests were performed to evaluate the relationship between the factors significantly associated with knowledge and acceptance after chi-square tests. A *p-value* less than .05 was considered statistically significant.

## RESULTS

### Socio-demographic Characteristics of the Participants

A total of 210 Jinja District residents participated in the study and completed the study questionnaire with a 100% response rate. While the majority were between the ages of 18 to 29 years (50%), female participants dominated the study (56.2%). The highest education level attained by most of the respondents was secondary school/high school and above (76.2%). The majority of the respondents were urban residents (52.4%) and mentioned others (39%) as their occupation. Details of the socio-demographic characteristics of the study participants are presented in Table 1.

**Table 1.** Socio-Demographic Characteristics of Study Participants (n=210)

Variable	Frequency	Percentage (%)
Gender		
Female	118	56.2
Male	92	43.8
Marital status		
Single	80	38.1
Married	93	44.3
Other (widowed/divorced/separated)	37	17.6
Education level illiterate/primary school	50	23.8
Secondary / high school and above	160	76.2
Occupation (n= 209)		
Farmer	58	27.6
Health worker	21	10
Student	24	11.4
Teacher	24	11.4
Other	82	39
Age		
18-29	105	50
30-39	60	28.6
40 and above	45	21.4
Residence		
Urban	110	52.4
Rural	100	47.6

### Knowledge of Participants Regarding COVID-19 Vaccine

82.9% of respondents knew that vaccination is one of the ways to protect ourselves from contracting COVID-19. However, 36.2% of the participants were unaware of the effectiveness of the COVID-19 vaccine. 78.1% also answered that taking an overdose of vaccine can be harmful to the body. Around 78% of the respondents knew that the COVID-19 vaccine is free in Uganda, and 56.7% stated that COVID-19 vaccine does not cause autoimmune diseases. 65.7% responded that infertility is not one of the side effects of the COVID-19 vaccine. Overall, 45.2% of the respondents had adequate knowledge of the COVID-19 vaccine (Table 2).

**Table 2.** Participants' Knowledge on COVID-19 Vaccine (n=210).

Variable	Response	Frequency	Percentage (%)
Vaccination is one of the ways to protect ourselves from being infected with COVID-19.	Yes	174	82.9
	No	36	17.1
Only one dose of COVID-19 vaccine is enough for the body to fight against the COVID-19 virus.	Yes	68	32.4
	No	142	67.6
The COVID-19 vaccine is harmless, anyone aged 18 and above, including pregnant women can have it.	Yes	130	61.9
	No	80	38.1
One of the known side effects of the COVID-19 vaccine is infertility.	Yes	72	34.3
	No	138	65.7
COVID-19 vaccine ıs effective ?	Yes	134	63.8
	No	76	36.2
Even if there is a vaccine for COVID-19, other preventive measures are also very important.	Yes	160	76.2
	No	50	23.8
Using an overdose of COVID-19 vaccine can be harmful to our body.	Yes	164	78.1
	No	46	21.9
Vaccination against COVID-19 increases autoimmune diseases.	Yes	91	43.3
	No	119	56.7
The COVID-19 immunization program in Uganda started in March 2021.	Yes	121	57.6
	No	89	42.4
The COVID-19 vaccine is free in Uganda.	Yes	163	77.6
	No	47	22.4
Knowledge level of the participants	Inadequate	30	14.3
	Moderate	85	40.5
	Adequate	95	45.2

Apart from the education level of the participants (*p=.004*), no other significant difference was found in terms of knowledge level of COVID-19 vaccine for all the other demographic data (Table 3).

**Table 3.** Factors Associated with COVID-19 Vaccine Knowledge (n=210)

Factor	Category	Knowledge level						*P*
		Insufficient		Moderate		Sufficient		
		N	%	n	%	n	%	
Gender (n=210)	Female	12	10.2	47	39.8	59	50.0	.102
	Male	18	19.6	38	41.3	36	39.1	
Residence (n=210)	Urban	12	10.9	44	40	54	49.1	.271
	Rural	18	30.0	41	41	41	41.0	
marital status (n=210)	single	10	12.5	35	43.8	35	43.8	.755
	married	15	16.1	33	35.5	45	48.4	
	Other (widowed, divorced, separated)	5	13.5	17	45.9	15	40.5	
Education level (n=210)	illiterate/primary school	13	26	23	46.0	14	28	.004
	Secondary/high school and above	17	10.6	62	38.8	81	50.6	
Occupation (n=209)	Farmer	11	19.0	22	37.9	25	43.1	.574
	Health worker	1	4.8	6	28.6	14	66.7	
	Student	3	12.5	11th	45.8	10	41.7	
	Teacher	3	12.5	8	33.3	13	54.2	
	Other	12	14.6	37	45.1	33	40.2	
Age (n=210)	18-29	12	11.4	49	46.7	44	41.9	.125
	30-39	12	20	16	26.7	32	53.3	
	40 and above	6	13.3	20	44.4	19	42.2	

Logistic regression analysis for the statistically significant variables in bivariate analysis showed that participants with secondary/high school and above level of education were 3 times more likely to have adequate knowledge of the COVID-19 vaccine than the illiterate or those with primary school education level (OR = 2.64; 95% CI; 1.32-5.26, *P=.006*) (table 4).

**Table 4.** Logistic Regression Analysis of Sociodemographic Characteristics Associated with COVID-19 Vaccine Knowledge

Variable	OR	95% CI	P-value
Education level (n=210)			
illiterate/primary school	1		
Secondary/high school & above	2.64	1.32-5.26	.006

**Figure 1.**
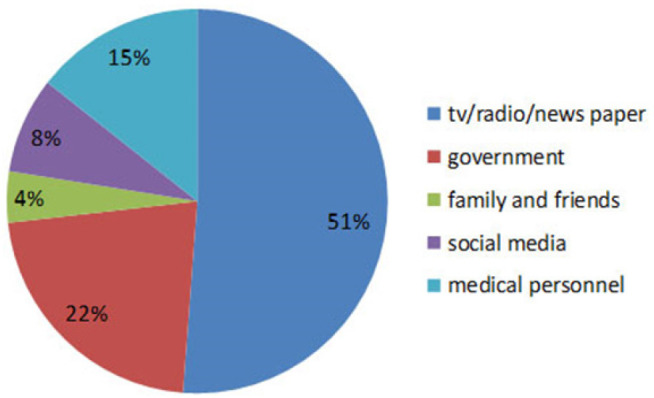
COVID-19 Vaccine Related Source of Information (n=209)

As shown in Figure 1, the source of information for most people about the COVID-19 vaccine is TV/radio/newspaper (51%); 22% government; 15% medical personnel; 8% social media; while only 4% have received information from family and friends. (Figure 1).

### COVID-19 Vaccine Acceptance

**Figure 2.**
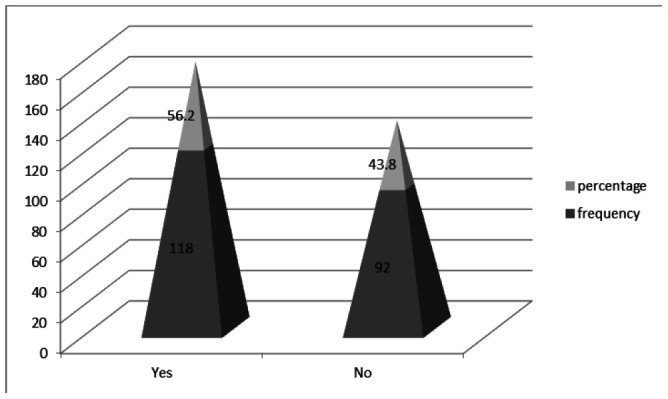
Acceptance of COVID-19 Vaccine among Jinja District Residents (n=210)

In this study, approximately 56.2% of the participants were willing to be vaccinated, while 43.8% were unwilling (figure 2).

**Figure 3.**
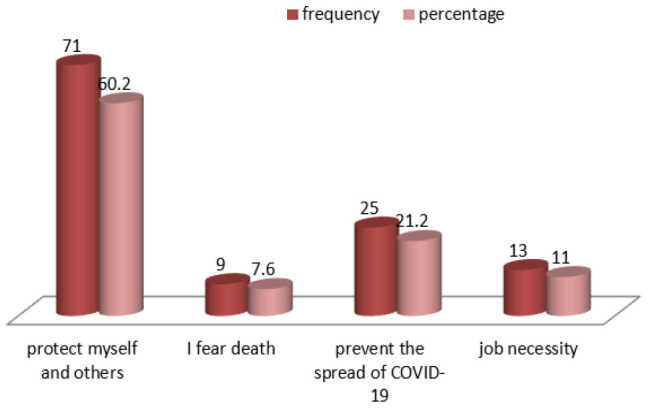
Reasons for COVID-19 Vaccine Acceptance

The reason for accepting the vaccine by 60.2% of the participants was to protect themselves and others, 21.2% to prevent the spread of COVID-19, 7.6% cited fear of death and for 11% it was a job necessity. (figure 3).

**Figure 4.**
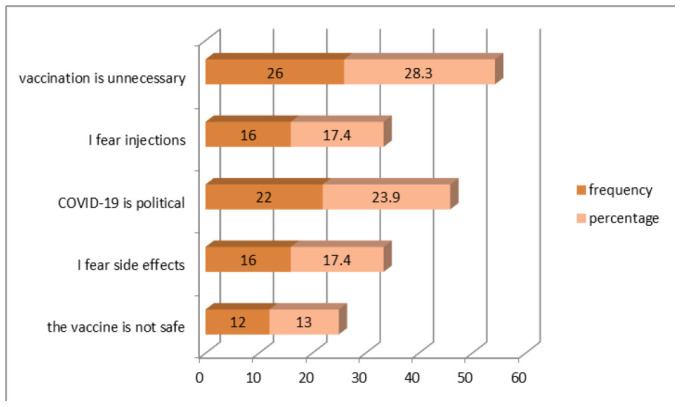
Shows the Reasons for Hesitation towards COVID-19 Vaccine

Of the 92 participants who did not want to be vaccinated, 28.3% stated that COVID-19 vaccination was unnecessary and 17.4% feared side effects. In addition, 23.9% said that COVID-19 is political, 17.4% feared injections, and 13% said they were worried about vaccine safety (figure 4).

In bivariate analysis; Education level (*p=.003*) and occupation (*p< .001*) were significantly associated with the acceptability of the COVID-19 vaccine. No significant association was found with gender (*p= .845*), residence (*p= .74*), marital status (*p= .398*), age (*p= .77*), and COVID-19 vaccine information source (*p=.368*) (table 5).

**Table 5.** Factors Associated with COVID-19 Vaccine Acceptance (n=210)

Factor	Category	Level of acceptance				*P*
		Yes		No		
		N	%	N	%	
Gender (n=210)	Female	67	56.8	51	43.2	.845
	Male	51	55.4	41	44.6	
Residence (n=210)	Urban	63	57.3	47	42.7	.74
	Rural	55	55	45	45	
Marital status (n=210)	Single	41	51.2	39	48.8	.398
	Married	57	61.3	36	38.7	
	Other (widowed, divorced, separate)	20	54.1	17	45.9	
Education level (n=210)	illiterate/primary school	19	38	31	62	.003
	Secondary/high school & above	99	61.9	61	38.1	
Occupation (n=209)	Farmer	21	36.2	37	63.8	<.001
	Health worker	17	81	4	19	
	Student	9	37.5	15	62.5	
	Teacher	17	70.8	7	29.2	
	Other	53	64.6	29	35.4	
Age (n=210)	18-29	53	50.5	52	49.5	.770
	30-39	41	68.3	19	31.7	
	40 and over	24	53.3	21	46.7	
COVID-19 vaccine information source (n=209)	TV/radio/newspaper	55	51.4	52	48.6	.368
	Government	27	58.7	19	41.3	
	medical personnel	20	66.7	10	33.3	
	family and friend	4	44.4	5	55.6	
	Social media (whatsup, facebook, twitter)	12	70.6	5	29.5	

Logistic regression analysis showed that participants with secondary/high school and above education level were approximately 3 times more likely to agree to be vaccinated than those who are or with primary school level of education (OR= 2.7; 95% CI; 1.38-5.10, *p= .004*). Participants whose occupation was farming (OR= 0.31; 95% CI; 0.15-0.63, *P=.001*) or students (OR=0.33; 95% CI; 0.13-0.84, *P=.021*) were significantly less likely to accept the COVID-19 vaccine (Table 6).

**Table 6.** Logistic Regression Analysis of Sociodemographic Characteristics Associated with COVID-19 Vaccine Acceptance

Variable	OR	95% CI	*P-value*
Education level (n=210)			
Illiterate/primary school	1		
Secondary/high school & above	2.7	1.38-5.10	.004
Occupation (n=209)			
Other	1		
Teacher	1.03	0.38-3.47	.573
Student	0.33	0.13-0.84	.021
Health worker	1.33	0.41-7.26	.161
Farmer	0.31	0.15-0.63	.001

In addition, the effect of participants' knowledge on vaccine acceptance was determined. The results showed that there was a weak, and positively significant correlation between participants' knowledge and vaccine acceptance (r= 0.248, *p= .002*) (table 7).

**Table 7.** Relationship between Knowledge and Acceptance of COVID-19 Vaccine.

variable	Correlation Coefficient	P-value
Knowledge – Acceptance	0.248	.002

## DISCUSSION

In the fight against the COVID-19 pandemic, which is claiming numerous lives and wreaking havoc on economies, vaccination stands out as the most effective public health solution of all time. By the end of September 2021, most developed countries had achieved the World Health Organization's 10% target for vaccinating their general population, with the notable exception of Africa. The low vaccination rate on the African continent may be attributed to inadequate vaccine supply, limited accessibility, or vaccine hesitancy.

This research report presents findings from a study conducted in Jinja District to evaluate residents' knowledge and acceptance of the COVID-19 vaccine. I believe that the current study's findings will assist relevant authorities in developing increased awareness and health education programs concerning the COVID-19 vaccine in Jinja DistrictIn this study, it was found that adequate level of knowledge among the general population was 45.2%. The low level of adequate knowledge about COVID-19 vaccine among the study population may be attributed to inconsistencies in the quality of information provided to the public. Another contributing factor is the use of inappropriate means of communication, such as social media, by the responsible bodies when disseminating information about the COVID-19 vaccine, despite the majority of the local population relying on radio and television as their primary sources of information. The study finding was very low compared to a similar study conducted in India (82%) and Ethiopia (74%).^17,2^ Differences in methodology, participants' socio-demographic characteristics, and the conveyance of information about the COVID-19 vaccine could be the possible reason for the low level of adequate knowledge observed. The present study findings are consistent with another study conducted in Ethiopia.^19^

Most of the participants knew that vaccination is one of the ways to protect humans against covid-19. These findings were similar to those of Oman.^21^ In addition, many of the participants knew that the COVID-19 vaccines were effective, and agreed that despite vaccine availability, other preventive measures are also important in preventing COVID-19; this is similar to the findings of an Ethiopian study.^19^ Also, most of the participants knew that the COVID-19 vaccines were free, but only half of them knew when the vaccination program started. This was also observed in a study done in India.^17^

In this study, participants with secondary/high school education and above were more likely to have a high level of COVID-19 vaccine related knowledge. This could be attributed to their higher chances of accessing information about the COVID-19 vaccine especially on the internet. A similar finding was observed in Ethiopia, India, and Bangladesh.^9,17,1^

Additionally, the present study findings are similar to results reported by a COVID-19 (not vaccine) awareness and knowledge study among the general population of Syria.^20^ Surprisingly, the knowledge level of the participants was not correlated with either age or gender. The findings align with those of studies conducted in Bangladesh and Ethiopia.^1,19^ Residence was also found to be among the factors that did not influence the participant's level of knowledge about the COVID-19 vaccine, a similarity noted in a study conducted in India.^17^

In contrast to the findings from Oman, Central Uganda and Bangladesh, where the main source of information on COVID-19 vaccine for the participants was social media, most of the participants in this study received information about the vaccine from radio/television/newspaper.^21-22,1^ The disparities in findings may stem from the fact that these other studies were conducted online. Government and other public health officials should regularly utilise television and radio as a means of communication to enhance the general population's awareness of the COVID-19 vaccine. Additionally, it is essential to provide local people with more detailed, clear and accurate information about COVID-19 vaccine. The current study revealed that the general population's willingness to be vaccinated against COVID-19 was 56.2%. This vaccine acceptability rate is low for achieving herd immunity by covering at least 60% of a 100% efficacy vaccine for life-long protection.^23^ The low acceptance of vaccines among the study population may be related to the lower incidence of deaths from the COVID-19 virus in the country. In addition, the global Covid-19 infodemic may have played a role in increasing hesitancy towards vaccines.^24^ As observed in this study, if the acceptance and vaccination rate remain low, the pandemic will adversely affect not only the health of the general population, but also the country's economy. This finding was consistent with findings of studies conducted in western Uganda, Greece, and Oman.^25,7,21^ However, the finding was lower than that reported in Ethiopia (62.6%), India (88%), Australia (89.9%), United Kingdom (71%), and USA (69%).^2,17,26-28^ It was higher than that reported in Turkey (41.2%), Hong-Kong (34.5%) and Jordan (37.4%).^29-30,18^ The difference in the findings can be explained by the differences in socio-demographic characteristics of the respondents, materials and methods used in the study, and the availability and accessibility of the vaccine. Factors related to the health system may also be contributing to this difference.

Most of the participants in this study were willing to get vaccinated because they wanted to protect themselves and others. Similar findings were found in a global cross-sectional study of knowledge, attitude and acceptance of the COVID-19 vaccine.^16^ Although studies from Hong-Kong, India, Turkey, Greece, Jordan, Oman, and Sub-Saharan Africa reported fear of side effects and vaccine safety as the main causes of vaccine hesitancy, the current findings were somewhat different.^30,17,29,7,18,21,31^ Most of the study participants reported vaccination being unnecessary as the main cause of vaccination hesitation; Their response may be related to lack of adequate knowledge on COVID-19 vaccines. Other reasons for vaccine hesitation reported in our study are; vaccines not being safe, fear of side effects, fear of injections, and COVID-19 being political.

This research found that individuals with higher levels of education were more likely to be vaccinated than those with lower levels of education. This situation was different from that of a study in Sub-Saharan Africa, Greece, India and Oman.^31,7,17,21^ This was consistent with the findings in Turkey, United Kingdom, Australia, Western Uganda and Ethiopia.^29,32-33,2^ Besides education level, individual's occupation also had an impact on vaccine acceptance, that is to say farmers and students were significantly less likely to be vaccinated. In accordance to this, interventions and policies to increase vaccine acceptance in the region should primarily target farmers, students, and individuals with a low level of education. No significant difference in the vaccine acceptance rate according to age and marital status was observed. Similar findings have been reported in Turkey, India and Central Uganda.^29,17,22^ Another factor that had no effect on acceptance of the COVID-19 vaccine is gender. These study findings were in contrast to those from Western Uganda, Jordan, Hong-Kong, Oman, and Turkey.^25,18,30,21,29^

There was a positive and significant correlation between the general population's intention to accept the vaccine and the level of knowledge. This result is consistent with a study done in India and Greece.^17,7^ The present study findings suggest that adequate awareness of the COVID-19 vaccine will increase the uptake of the COVID-19 vaccine in the general population. In addition, public awareness campaigns for specific community needs have proven effective in increasing vaccination rates for other epidemics.^26^

## CONCLUSION

The general population of Jinja District demonstrated a low level of adequate knowledge and acceptance towards COVID-19 vaccine. To address this, more public awareness campaigns on the topic must be conducted using radio and television as means of communication. This study's findings can be utilised by organizations such as the WHO, CDC Africa and the country's public health officials to understand the obstacles to the COVID-19 mass vaccination program. This understanding can guide the implementation of necessary interventions to address the challenges. Furthermore, our study findings can serve as a reference for other researchers.

### Limitations of the Study

The study was conducted in a small geographic area (Jinja District), meaning that the findings cannot be generalised to the entire country. Therefore, it is appropriate to advocate for additional similar studies to be carried out in other regions of the country. Secondly, the study utilised self-administered surveys, which may have impacted the reliability of the results. Lastly, no internal validity assessment was performed for the questionnaire.
